# Fine Motor Precision Tasks: Sex Differences in Performance with and without Visual Guidance across Different Age Groups

**DOI:** 10.3390/bs10010036

**Published:** 2020-01-16

**Authors:** Liudmila Liutsko, Ruben Muiños, Josep Maria Tous Ral, María José Contreras

**Affiliations:** 1 Faculty of Psychology, MSU, 125009 Moscow, Russia; 2 Radiation Department, ISGlobal, 08003 Barcelona, Spain; 3 Department of Basic Psychology I, UNED, 28040 Madrid, Spain; 4 Psychological Studies, UOC, 08018 Barcelona, Spain; 5 Dep. Personality, Assessment and Psychological Treatments, UB, 08035 Barcelona, Spain; josepmaria.tous@esrp.net

**Keywords:** fine motor precision, vision, proprioception, sex differences, individual differences, personality

## Abstract

Previous studies have reported certain sex differences in motor performance precision. The aim of the present study was to analyze sex differences in fine motor precision performance for both hands under different test conditions. Fine motor tasks were performed by 220 Spanish participants (ages: 12–95), tracing over the provided models – lines of 40 mm for both hands, two sensory conditions (PV—proprioceptive-visual; P—proprioceptive only) and three movement types (F—frontal, T—transversal, and S—sagittal). Differences in line length (the task focused on precision) were observed through MANOVA analysis for all test conditions, both sexes and different age groups. Sex differences in precision were observed in F and T movement types (statistically significance level and higher Cohens’ d were observed in condition with vision). No statistically significant differences were observed for both hands and sensory conditions in sagittal movement. Sex differences in fine motor precision were more frequently observed in the PV sensory condition in frontal movement and less in sagittal movement.

## 1. Introduction

Recent studies on sex differences in motor precision are scarce, with most studies found in a Google Academic search having been carried out in the past century (1980s–1990s). The suggested model that best fits such differences (including sex differences) is a biopsychosocial model that optimally combines both nature and nurture approaches [1,2], where Halpern [2] precisely pointed out that “differences are not deficiencies”. Thus, studying and describing sex differences is similar to that of any other individual difference or personality, way of existing or being. For example, it was found that men are better in some spatial tasks’ performances (mental spatial rotation) compared to women; whereas women were found to be better in fine motor skills tests [2].

Going back in history, Heymans [3] was one of the first to study sex/gender differences, noting that schoolboys were considered to be more active (difficulty in remaining seated quietly) and with preference for active games, whereas girls were characterized by quiet behavior (in the motor, not emotional sense). Such activeness was seen in boys as more aggressive behavior compared to girls. However, as they grew older, women were more active in comparison with men: gesticulating more, jumping up from their seats and walking in the apartment [3]. According to male experts, men enjoy practicing sport more frequently than women (walking, biking, skating, billiards, hunting, etc.); although female experts considered that men and women enjoyed sport activities similarly [3]. The most important characteristic of motor behavior in women in comparison to men (according to Heymans) is their higher emotionality.

Bagrunov’s [4] study (341 men and 268 women) showed that men had a prevalence over women in the integration of two psychomotor parameters: speed and precision, although women showed better training effects (psychomotor functions in women were easier to train). Men also demonstrated higher performance in new sensorimotor tasks, and women in those that were more stereotyped. In general, men had greater species variability in the motor area, whereas women had higher individual variability. Women were more original in their movements and, in some situations, did not behave like their usual selves. Allahverdov [5] arrived at a similar conclusion: a reflection of everyday views about women as inconstant and unpredictable in comparison to men. Danilova [6] concluded that men had a greater ability for motor skills such as aiming, catching, and throwing, whereas women were better in tasks where precision and fine hand ability are needed.

Traditionally, men have shown a prevalence in gross motor performance and women in fine motor performance, due to gender roles and the work they are accustomed to carrying out more frequently. However, with time, these differences have become more insignificant due to emancipation processes and changes in male/female roles in some societies (especially in western societies) [7]. Moreover, sex differences in motor behavior could reflect, as per previous studies, the existing differences in personality [8] and individual cultural differences [9,10].

Rose [11] found that for any extreme situation (related to vestibular intellectual, as well as emotional loads, as in the case of an exam), women reacted more strongly than men but recovered faster to basal levels. In another study on motor precision of both hands in aiming ([11]; N = 104, age 2–30 years old), alternative periods of prevalence between both sex performances were found depending on age: before 7 years old in favor of boys; from 10 to 18–25 in favor of girls/women. The precision of the left hand was always higher in comparison with men, with the exception of ages 20 to 21. In this study, the age differences, hand asymmetry, and variability were greater in men with sharp changes, while for women, these parameters changed more smoothly. Women showed more symmetrical precision in movements for both hands, while men performed more asymmetrically (with dominance for the right hand in precision). These findings were congruent with Ananiev’s [12] scheme that differentiated between sexes, according to which men needed to use additional adaptive mechanisms (asymmetry) while women passed with basic ones (symmetry). In general, women were found to have less asymmetry between both hands performances in fine motor tasks; though changes in symmetry vs. asymmetry occurred depending on age, being more “asymmetrical” during the adolescent period for both men and women [13].

Revising further recent research, some findings are unclear or even contradictory in relation to sex differences and motor precision, velocity, and asymmetry and these parameters seem to be age-related. In both, primary school (8–12) and high school students (15–18), girls showed prevalence in handwriting short copy tasks vs. boys, while in another longitudinal study (7–11), copywriting tasks did not reveal any significant sex differences [14]. A male advantage in motor learning rather than in motor performance was found in male adolescents compared to female adolescents [14].

Thus, the complexity of comparing the results obtained by different studies consists not only of the important factor of age, as shown by the above literature review, but also of other aspects, such as the type of tasks and sensory conditions used in those tests. Sometimes, other factors can also influence fine motor performance in men and women, such as socio-economical status [15], individual [16], and cultural differences [10] among others; these factors should be considered in interpretations whenever possible. In addition, the important role of proprioception for motor tasks and perception of space was observed in previous studies, together with the crucial role of the integrative system vision with proprioception [17]. Moreover, since cognitive performance in spatial tasks is the main difference in sex performance, especially underling the way both groups perform and not the final results [18], the motor and cognition performances could be interrelated.

The aim of the present study was to explore sex differences in fine motor precision performance tasks in both hands with the use of new technologies (digitalized), with different age subgroups and test conditions. Both tested sensory conditions – with visual guidance (PV—proprioceptive-visual) and without (P—proprioceptive only) – have input from a proprioceptive sense (in the first one, integrated with vision).

The questions of the study are as follows:

1. Are there any sex differences in fine motor precision across the entire sample in different age subgroups? Our hypothesis, as per previous studies, is that there should be some differences in fine motor precision in men and women.
2. Are there any sex differences in fine motor precision across the different test conditions (movement types: F—frontal; T—transversal, and S—sagittal) and sensory conditions (PV—proprioceptive-visual and P—proprioceptive only)? Would any movement type/s or sensory condition be more sensible to sex differences?

## 2. Materials and Methods

### 2.1. Participants and Data Analysis

The Proprioceptive Diagnostics of Temperament and Character (DP-TC in Spanish, [19]) test was performed by 220 Spanish participants with normal or corrected to normal vision from the general population (ages: 12–95, 63% men). Participants were self-reported as healthy people who were not undergoing any medical treatments. All participants took part voluntarily, were informed about the aims of the research and gave their consent prior to their inclusion in the study. The study was conducted in accordance with the Declaration of Helsinki, and the protocol was approved by the Ethics Committee of RTI2018-098523-B-I00 and PhD thesis approval committee by the University of Barcelona.

### 2.2. Tools

**Figure 1:**
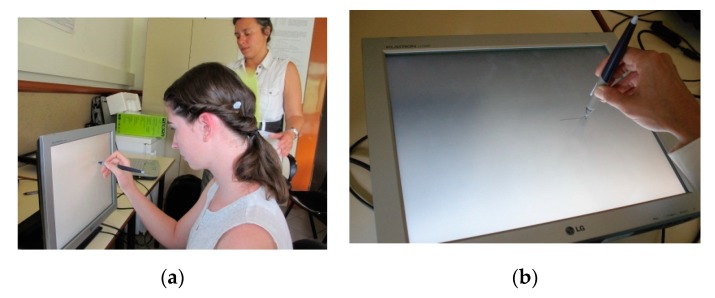
Lineograms test: in frontal movement type (**a**) and in transversal movement type (**b**).

Proprioceptive diagnostics [19] were used to register and measure the graphical movements – line tracings in different test conditions (Figure 1).

### 2.3. Stimuli, Observable Variables, and Data Analysis

The stimuli were 40 mm lines (Lineograms) represented under different test conditions. Precision in fine motor precision was measured under different test conditions, three movement types (F—frontal, T—transversal, and S—sagittal), both hands (ND—non-dominant and D—dominant) and two sensory conditions (PV and P), as observable variable LL—line length – in men and women and at different age groups. Thus, the complete model was described by variables of precision (LL—line length) that depended on three factors of test conditions (MT—movement type, SC—sensory condition, and hand) and sex (in different age subgroups). For the analysis, the participants were grouped into four age groups, representing different stages of developmental and professional activities: (1) 12–17—adolescents (scholars) (N = 41, 55% men); (2) 18–29—young adults (mainly students) (N = 63, 68% men); (3) 30–64—adults (mainly professional workers) (N = 72, 65% men), and (4) 65–95—elder group age (mainly retired) (N = 44, 42% men). The statistical analysis (descriptive and MANOVA with Bonferroni post-hoc analysis) was performed using SPSS.

### 2.4. Instructions

The instructions to participants were to start each subtest (with visual guidance) by tracing the model line as precisely as possible (repetitive movements without interruptions) and to continue doing them (without visual guidance) until the signal “stop” was given. The acting hand was not supported (not touching the screen). The participants were asked to sit in an upright position, without leaning, maintaining body balance, and without crossing their legs [19].

## 3. Results

**Figure 2:**
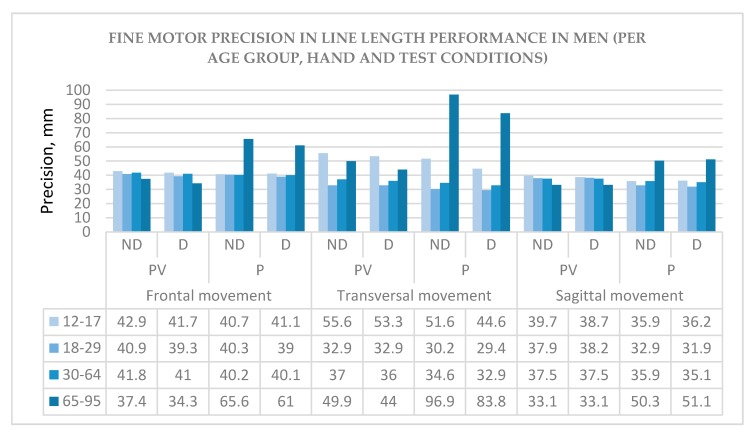
Fine motor precision in men/Note: The model line length is 40 mm.

**Figure 3:**
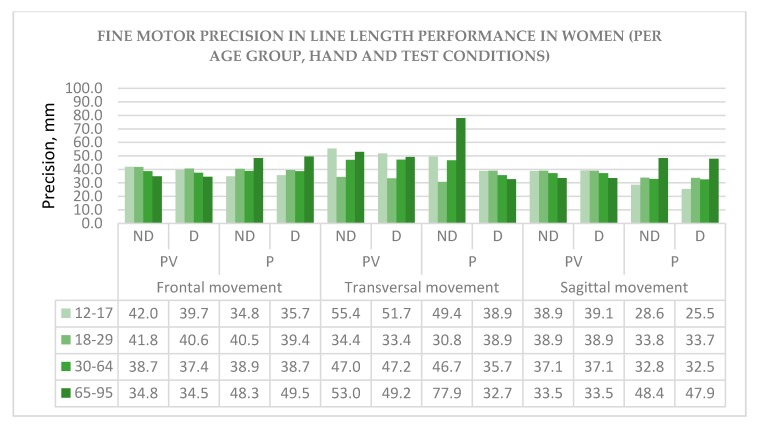
Fine motor precision in women. Note: The model line length is 40 mm.

The descriptive statistics for fine motor precision is given for men (Figure 2) and women (Figure 3) depending on age group (12–17, 18–29, 30–64, and 65–95), hand (ND—non-dominant and D—dominant), and test conditions: Movement type (frontal, transversal and sagittal) and sensory condition (PV—proprioceptive-visual and P—proprioceptive only).

The MANOVA analyses of sex differences in the graphical performance of line length size under different test conditions are shown in Table 1.

No statistically significant difference between both sex subgroups was found in sagittal movement. The statistically significant differences in fine motor precision were shown for frontal movement (with the exception of the dominant hand and P-only sensory condition, where a statistically significant level was not reached, *p* = 0.62) and the transversal movement type (only in PV sensory condition with visual guidance) (Table 1). The interaction of sex by age group was significant only in precision and PV sensory condition in frontal (*p* < 0.045 in ND and *p* < 0.001 in D hands) and transversal movement types (*p* < 0.001 for both hands).

## 4. Discussion

The results of our study, in general, would not confirm Danilova’s [6] idea that women have higher precision compared to men in fine motor movements, as per our results, it is only partially true (for some movement types, test conditions, and age groups). The age had a crucial role in changes in fine motor precision, similarly to previous studies although these covered fewer age periods (<30 years old) [11]. In the majority of cases, men drew longer lines compared to women. Men had a tendency to overperform the model line length (40 mm) and women showed a tendency to underperform it; but the absolute precision—the precision bias without taking into account a sign—was better or worse, alternatively changing in favor of one or the other sex subgroup depending on test conditions and age. This may reflect individual differences between sexes, similar to those found by other researchers in personality [8]. Each movement type in the proprioceptive sensory conditions is associated with a different personality dimension as per Mira’s suggestion [20]. For example, frontal movements represent a vital tonus (from pessimism or little energetical level to optimism or high energetical level). Transversal movements represent the balance between attention paid to internal and external words; and a sagittal movement, from submission to dominance [9,14,19,20].

The statistically significant differences found for the precision performance between representatives of both sexes in the non-dominant hand that reflect more constitutional or biologically determined indicators [19] favored both men and women, depending on the age group in the PV sensory condition in frontal movement. However, sex differences were attributed more to the opposite direction of average group bias; thus resulting in different ways of approximation to the model line.

Since the P-only sensory condition performance underlies individual differences and personality, as also suggested by Tous and colleagues’ [19] work, differences such as outperforming line length in men and underperforming in women suggest interpretation of the balance between excitability and inhibition, and, in the present study, a more inhibited nature of girls of 12–17 compared to the same age boys.

Among the present study’s limitations, the self-reported vision and health state can be mentioned. In both cases of those who considered their vision normal and those who wore glasses to correct vision to normal, actual vision was not verified before the study.

As there are very few studies carried out in this direction, the findings represented here can contribute to a greater understanding of sex and age differences in fine motor tasks. These findings can also help to understand the relationship between two sensory modalities in performance: PV—proprioceptive-visual and P—proprioceptive only. Moreover, age-dependent trends are also important to see the evolution of precision in both sexes. For example, in frontal and sagittal movement types, at the older age group of 30–64, a tendency to underperform line length in the PV sensory condition, and outperform in the P-only condition can be observed and this trend is similar in men and women (Figure 2 and Figure 3). This inverted relationship could suggest the existence of compensatory mechanisms between the two sensory modalities and requires further study to confirm the hypothesis.

## 5. Conclusions

In this context, with regard to test conditions and age-dependent disclosure of the results, this is a pioneer study as far as we are aware. The sex differences reported by the current study in fine motor precision are linked to the average individual differences of both sex groups and could shed light on the different ways to perform and perceive between both sex subgroups in general. If the performance of both groups is compared with the model, the precision (being better or worse) alternates for one sex subgroup compared to the other, depending on the age group and test conditions. In general, men had a tendency to outperform the model line length and women showed a tendency to underperform it for the majority of the observed cases as per different test conditions. However, generally, more effects were observed according to age groups rather than sex.

## Figures and Tables

**Table 1:** MANOVA analyses results for the factor “sex”.

Test Conditions		MANOVA Results for LL (Line Length)			
MT	Hand	SC	*F*	*p*-Value	Cohen’s *d*
Frontal	ND	PV	**5.80**	0.017	0.38
		P	**6.24**	0.013	0.08
	D	PV	**4.26**	0.040	0.54
		P	3.52	0.062	0.02
Transversal	ND	PV	**15.68**	<0.001	0.66
		P	0.28	0.599	0.36
	D	PV	**15.80**	<0.001	0.63
		P	0.41	0.521	0.47
Sagittal	ND	PV	0.61	0.437	0.29
		P	2.10	0.149	0.05
	D	PV	0.19	0.665	0.15
		P	2.01	0.158	0.02

*Legend*: MT—movement type; SC—sensory condition; ND and D—non-dominant and dominant; PV—proprioceptive-visual, P—proprioceptive only. The statistically significant differences are in **bold**.

## Data Availability

The datasets used and analyzed during the current study are available from the corresponding author on reasonable request.
